# Proteomic profiles of unilateral cryptorchidism in pigs at different ages using MALDI-TOF mass spectrometry and in-gel digestion coupled with mass spectrometry (GeLC-MS/MS) approaches

**DOI:** 10.1186/s12917-020-02591-1

**Published:** 2020-10-02

**Authors:** Nathamon Yimpring, Sittiruk Roytrakul, Janthima Jaresitthikunchai, Narumon Phaonakrop, Sucheewin Krobthong, Gunnaporn Suriyaphol

**Affiliations:** 1 Biochemistry Unit, Department of Physiology, Faculty of Veterinary Science, Chulalongkorn University, 39 Henri-Dunant Road, Wangmai, Pathumwan, Bangkok, 10330 Thailand; 2 Proteomics Research Laboratory, National Center for Genetic Engineering and Biotechnology (BIOTEC), National Science and Technology Development Agency, 113 Thailand Science Park, Phahonyothin Road, Khlong Nueng, Khlong Luang, Pathum Thani, 12120 Thailand

**Keywords:** Cryptorchidism, In-gel digestion coupled with mass spectrometry (GeLC-MS/MS), Matrix-assisted laser desorption/ionization time-of-flight mass spectrometry (MALDI-TOF MS), Pig, Tumor necrosis factor receptor superfamily member 18 (TNFRSF18)

## Abstract

**Background:**

Cryptorchidism is a condition that occurs when one or both testes fail to descend into the scrotum. It is a common congenital disorder, causing economic loss in pig production. However, there have been only limited studies of differential protein expression profiles in undescended testes (UDTs) in the abdomen and descended testes (DTs) in cryptorchid pigs, especially at the peptidome and proteome levels. The present study aimed to analyze the peptidome of UDTs and DTs in unilateral cryptorchid pigs aged 1–2, 6, 15 and 20 weeks and in normal testes of healthy pigs aged 1–2 and 12 weeks, using peptide mass fingerprinting and three-dimensional principal component analysis (3D-PCA) with matrix-assisted laser desorption/ionization time-of-flight mass spectrometry, and to identify potential protein candidates, using in-gel digestion coupled with mass spectrometry (GeLC-MS/MS). Western blot analysis was used to verify protein expression. Protein sequence was affirmed by liquid chromatography–tandem mass spectrometry.

**Results:**

A PCA plot showed a discrete cluster for each sample group. Peptide mass fingerprints (PMFs) demonstrated unique peptide fragments in UDTs at different ages. A number of markedly expressed proteins from GeLC-MS/MS were identified, including the multifunctional tumor necrosis factor receptor superfamily member 18 (TNFRSF18), in DTs at 1–2 and 6 weeks and in UDTs at 15 and 20 weeks of age. Using western blot analysis, high expression of TNFRSF18 was observed in the UDTs at 15 weeks. Using the STITCH database, this protein was found to be related to apoptosis, corresponding to the previous report in the UDTs at the same age.

**Conclusions:**

The present study revealed the specific PMFs and clusters for porcine cryptorchidism, and a novel protein, TNFRSF18, associated with the disease mechanism. These results could provide further insights into the pathogenesis of the disease.

## Background

Cryptorchidism is a condition that occurs when one or both testes fail to descend into the scrotum, which can reduce fertility and increase the risk of testicular malignancies [1]. The prevalence of unilateral and bilateral cryptorchidism in pigs is 2.2 and 0.2%, respectively [2]. The undescended testis (UDT) in the abdomen interrupts normal spermatogenesis because of the high temperature and leads to an unpleasant boar taint in pork [3–5]. In normal pigs, testicular descent into the scrotum is complete by the time of birth [1]. Commercial fattening pigs are commonly castrated at the age of 7 days. A non-palpable testis in the scrotum at this age indicates that the disease is probably present, and pigs usually undergo surgery at the age of 6 weeks to remove the undescended testis [6]. The economic benefit of surgery versus cost (approximate 300 baht/nursery pig) and risk of surgery need to be critically evaluated. If the UDT is retained in the abdomen, prices of cryptorchid pigs at farms are cut by approximate 5 baht/kg of pig. For a farm with 10,000 sows, profits are reduced by 5,000,000 baht/year without including the inferior average daily gain and feed conversion ratio. Although animal breeders intensively select sire lines with no cryptorchidism in the great-grandparent (GGP) or grandparent (GP) generations, cryptorchidism often appears in the fattening pigs. The expression of protein markers could possibly be utilized not only for unveiling the aberrant mechanism underlying the disease, but also for selection of suitable sires from the GGPs and GPs, which might reduce the incidence of cryptorchidism in fattening pigs. To the best of our knowledge, only limited studies of the expression of proteins associated with porcine cryptorchidism have been reported, and a study of testicular proteomics of pigs with cryptorchidism has not been undertaken [6–8]. Hence, we decided to explore a panel of novel peptide and protein expression, using proteomic approaches.

High-throughput proteomics enables the study of proteins at a large scale [9]. Matrix-assisted laser desorption/ionization time-of-flight mass spectrometry (MALDI-TOF MS) has been used to reveal unique peptide mass fingerprints (PMFs), and clusters of each group can be identified using a three-dimensional principal component analysis (3D-PCA) scatterplot [10]. In-gel digestion coupled with mass spectrometry (GeLC-MS/MS), a one-dimensional sodium dodecyl sulfate–polyacrylamide gel electrophoresis (SDS-PAGE) coupled with liquid chromatography–tandem mass spectrometry (LC-MS/MS), has been used for identification of proteins [11]. The present study aimed to characterize PMFs and 3D-PCA scatterplots,, using MALDI-TOF MS, and to identify potential protein candidates associated with porcine cryptorchidism in UDTs and descended testes (DTs) in cryptorchid pigs, and normal testes (NTs), at the ages of 1–2, 6, 15 and 20 weeks, using GeLC-MS/MS. The results should be useful for veterinarians to better understand the pathogenesis of the disease and for swine breeders to select sires with low expression of protein candidates associated with the disease and to improve the efficiency of production systems.

## Results

### Peptide analysis results using MALDI-TOF MS

**Fig. 1:**
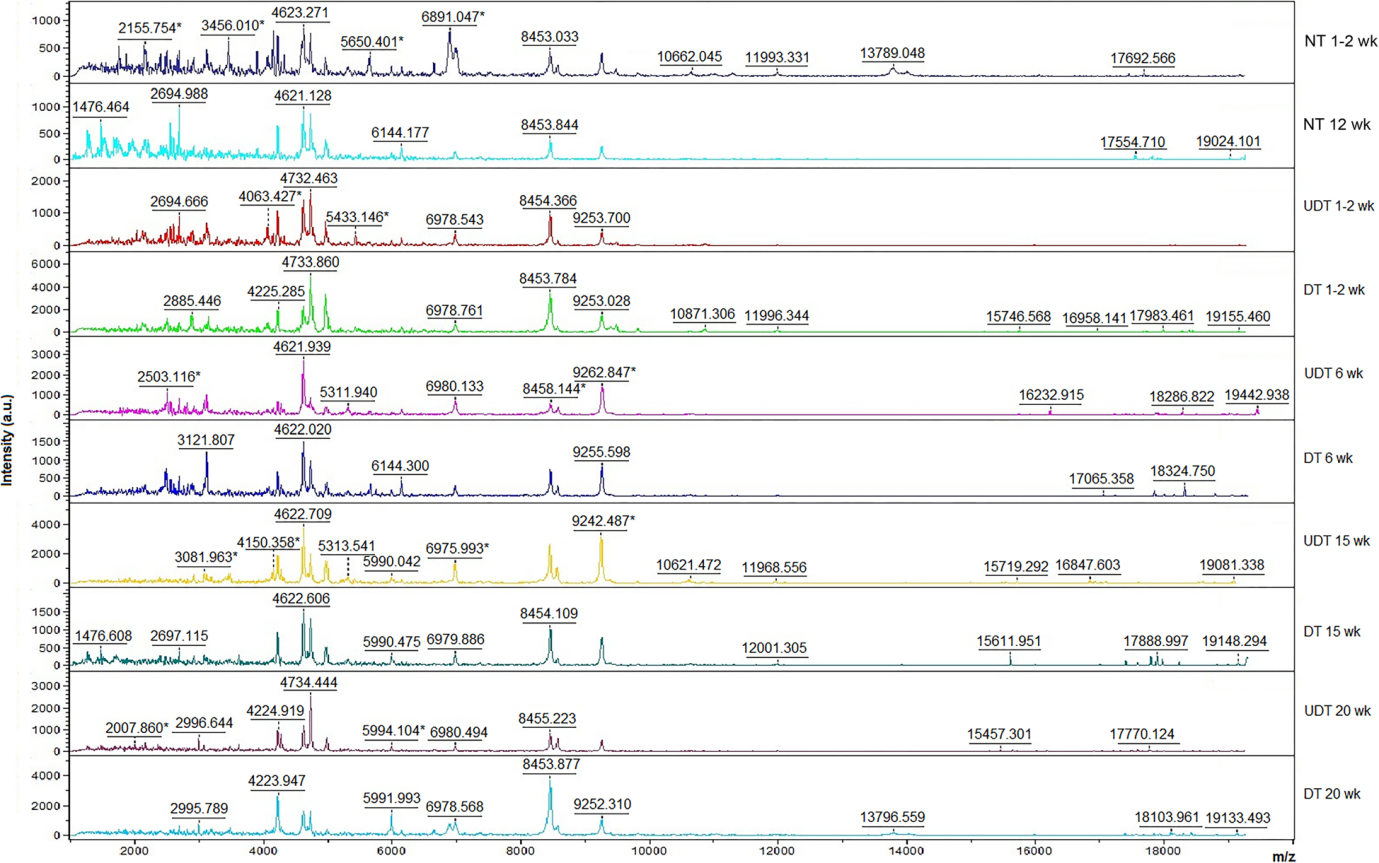
Peptide mass fingerprint of undescended testes in the abdominal cavity (UDT) and descended testes in cryptorchid pigs (DT) in unilateral cryptorchid pigs at the ages of 1–2, 6, 15 and 20 weeks (wk), and in normal testes of healthy pigs (NT) at the ages of 1–2 and 12 weeks. * indicates unique mass spectra peaks in each group

**Fig. 2:**
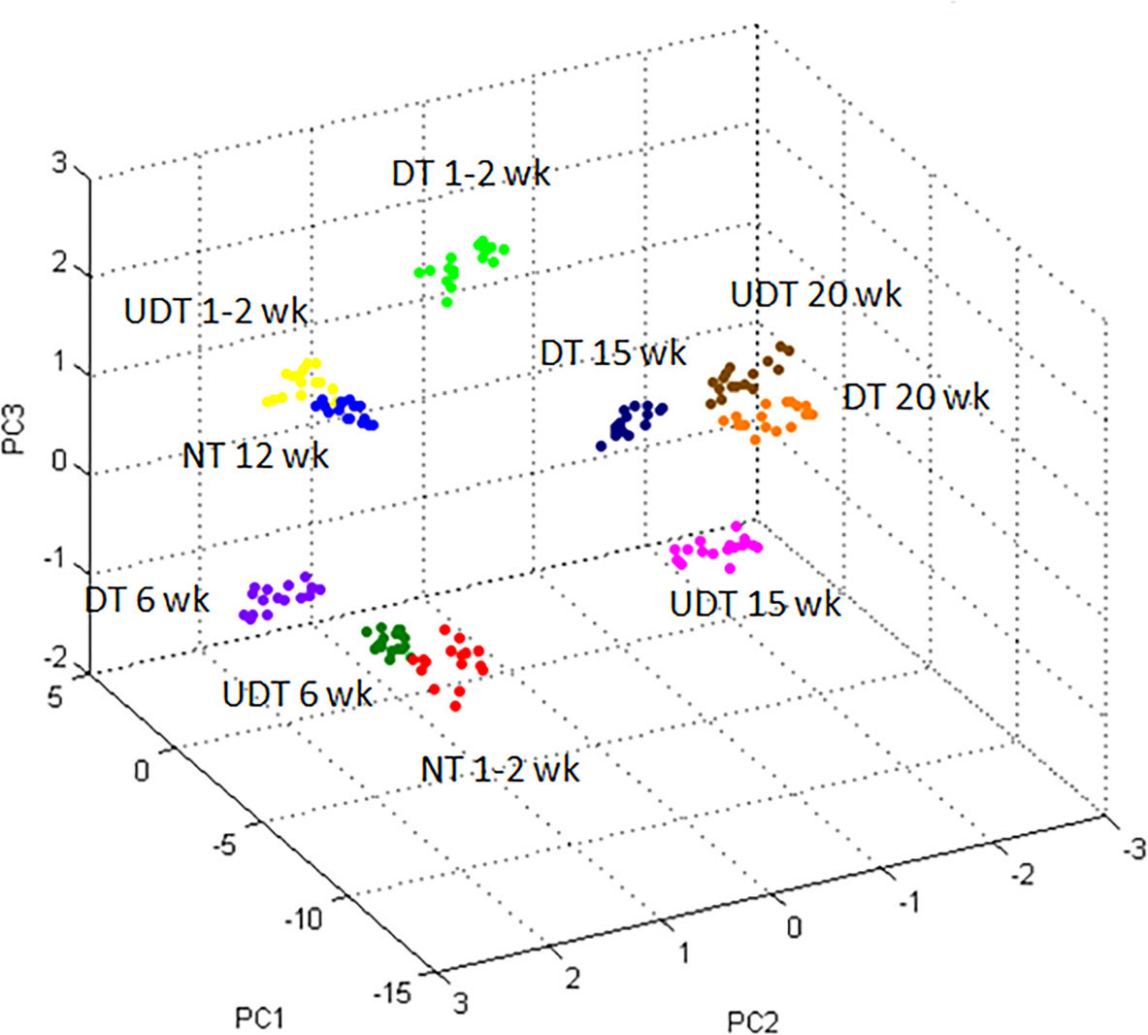
Three-dimensional principal component analysis scatterplot of undescended testes in the abdominal cavity (UDT) and descended testes in cryptorchid pigs (DT) in unilateral cryptorchid pigs at the ages of 1–2, 6, 15 and 20 weeks (wk), and in normal testes of healthy pigs (NT) at the ages of 1–2 and 12 weeks

PMF analysis revealed different patterns among sample groups in the range 1000–20,000 Da. Distinct peptide peak spectra were observed in UDTs at different ages and in NTs at 1–2 weeks of age (Fig. 1). The 3D-PCA scatterplot showed homogeneity and uniformity within the group. A cluster of UDTs at 1–2 weeks of age was close to that of NTs at 12 weeks of age, in addition to the close proximity of UDTs at 6 weeks of age and NTs at 1–2 weeks of age, and between UDTs and DTs at 20 weeks of age (Fig. 2). PMF and PCA scatterplot data indicated a high possibility of finding specific protein expression in each group, especially in the UDTs at 15 weeks and DTs at 1–2, 6 and 15 weeks of ages. In addition, different clusters of NTs and DTs at 1–2 weeks of age demonstrated that both groups were not interchangeable.

### GeLC-MS/MS results

**Fig. 3:**
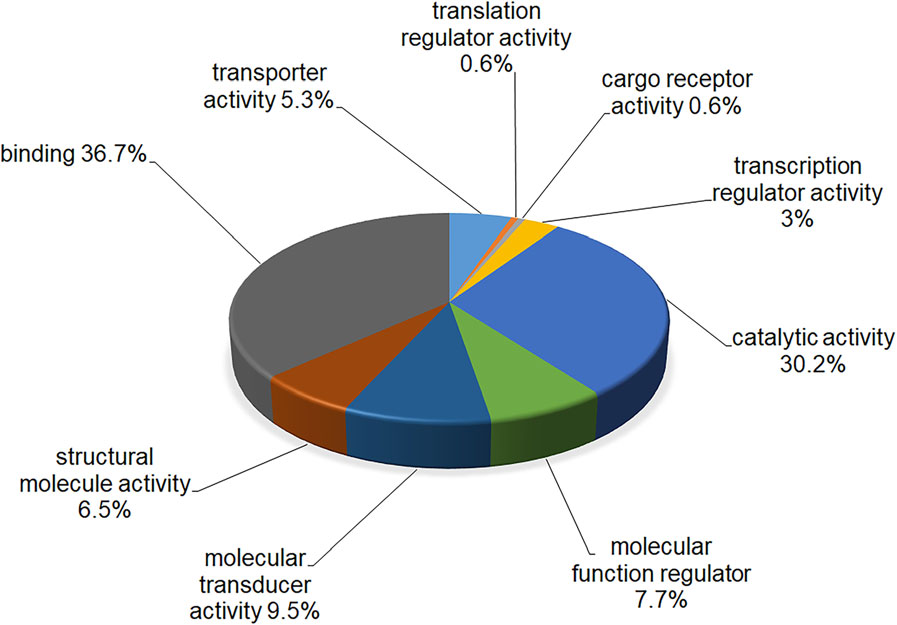
Pie chart showing the nine most common molecular functions of proteins differentially expressed in undescended testes in the abdominal cavity and descended testes in unilateral cryptorchid pigs at the ages of 1–2, 6, 15 and 20 weeks, and in normal testes of healthy pigs at the ages of 1–2 and 12 weeks

**Fig. 4:**
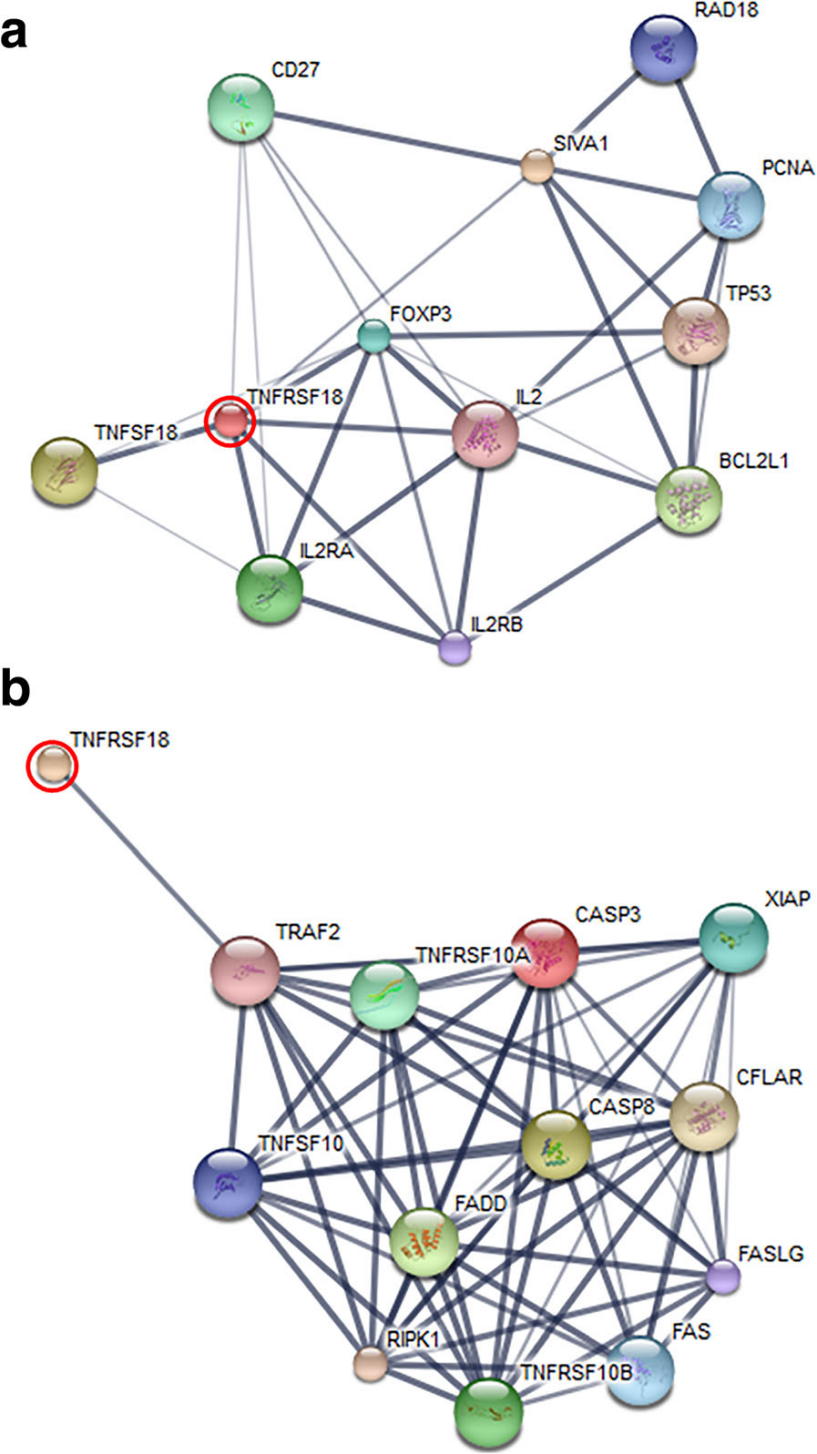
Relationship of tumor necrosis factor receptor superfamily member 18 (TNFRSF18) with apoptotic proteins (**a** SIVA1 and **b** caspases), analyzed by STITCH v. 5.0. Red circle: TNFRSF18. Abbreviations: BCL2L1, bcl-2-like protein 1; CASP3, caspase-3; CASP8, caspase-8; CD27, tumor necrosis factor receptor superfamily member 7; CFLAR, caspase-8 and FADD-like apoptosis regulator; FADD, FAS-associated death domain protein; FAS, Fas cell surface death receptor; FASLG, Fas ligand; FOXP3, forkhead box protein P3; IL2, interleukin-2; IL2RA, interleukin-2 receptor subunit alpha; IL2RB, interleukin-2 receptor subunit beta; PCNA, proliferating cell nuclear antigen; RAD18, E3 ubiquitin-protein ligase RAD18; RIPK1, receptor-interacting serine/threonine-protein kinase 1; SIVA1, apoptosis regulatory protein Siva; TNFSF10, tumor necrosis factor ligand superfamily member 10; TNFRSF10A, tumor necrosis factor receptor superfamily member 10A; TNFRSF10B, tumor necrosis factor receptor superfamily member 10B; TNFRSF18, tumor necrosis factor receptor superfamily member 18; TP53, tumor suppressor p53; TRAF2, TNF receptor-associated factor 2; XIAP, X-linked inhibitor of apoptosis protein.

From GeLC-MS/MS, a total of 4135 proteins were identified. Two hundred and thirty-two proteins were classified according to their molecular functions, using the PANTHER classification system (Fig. 3). The most common function was the binding of, or interaction with, molecules, whereas catalytic activity was ranked second. The relative expression levels of divergent proteins in UDTs, DTs and NTs at different ages are shown as log_2_ intensities in Supplementary Table S1 [12]. Groups of proteins differentially expressed in UDTs, DTs and NTs at different ages were revealed. Some proteins were shown to be expressed in UDTs at 1–2 weeks of age, such as tumor-associated calcium signal transducer 2, DNA repair protein complementing XP-A cells, breast and ovarian cancer susceptibility 1, sodium-driven chloride bicarbonate exchanger, vomeronasal type-2 receptor 26, 40S ribosomal protein S4-like, ankycorbin isoform X1, regucalcin-like, T-complex protein 1 subunit eta and calcium homeostasis endoplasmic reticulum protein. Tumor necrosis factor receptor superfamily member 18 (TNFRSF18) appeared in DTs at 1–2 and 6 weeks and in UDTs at 15 and 20 weeks of age (Supplementary Tables S1 and S2). This protein was shown to be associated with several apoptotic proteins, using the STITCH database (Fig. 4).

### Western blot analysis results

**Fig. 5:**
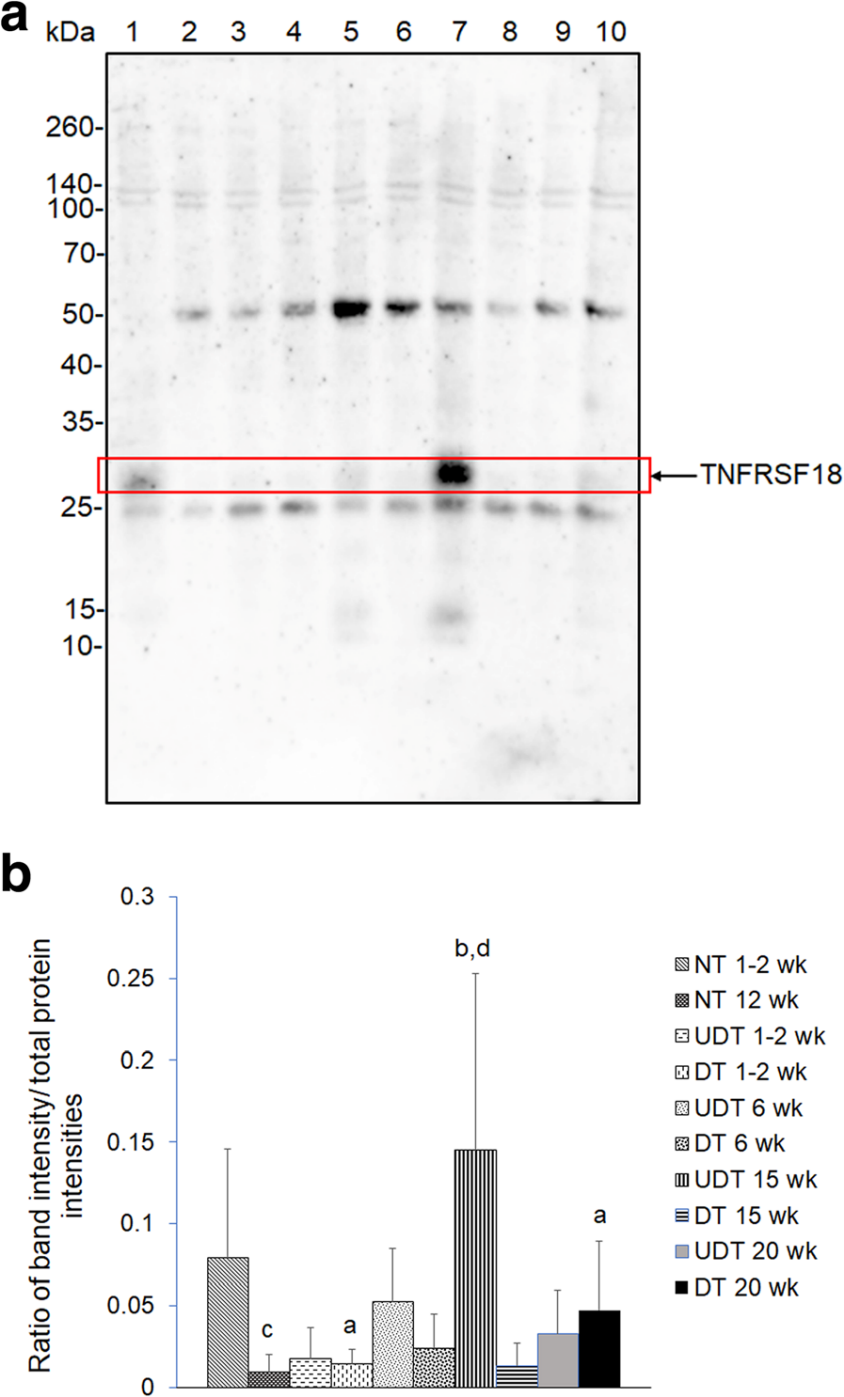
Western blot analysis of tumor necrosis factor receptor superfamily member 18 (TNFRSF18) of pigs with undescended testes (UDT) and descended testes (DT) at the ages of 1–2, 6, 15 and 20 weeks, and normal testes (NT) at the ages of 1–2 and 12 weeks. **a** Representative western blot for TNFRSF18 at 26 kDa. Lane 1, proteins from NT at the age of 1–2 weeks; lane 2, NT at the age of 12 weeks; lane 3, UDT at the age of 1–2 weeks; lane 4, DT at the age of 1–2 weeks; lane 5, UDT at the age of 6 weeks; lane 6, DT at the age of 6 weeks; lane 7, UDT at the age of 15 weeks; lane 8, DT at the age of 15 weeks; lane 9, UDT at the age of 20 weeks; lane 10, DT at the age of 20 weeks. **b** Bar graph of ratios of TNFRSF18 protein intensity to total blotted protein intensities in each lane; a-b and c–d denote a significant difference at *P* < 0.05 and *P* < 0.01, respectively

Western blot analysis revealed increased expression of TNFRSF18 in UDTs at 15 weeks of age compared with that in NTs at 12 weeks, DTs at 1–2 weeks and DTs at 15 weeks of age (Fig. 5 and Supplementary Fig. S1). LC-MS/MS was used to verify TNFRSF18 sequence. MAQHGAMGAFR fragment was found in TNFRSF18 by MS/MS.

## Discussion

This study showed that peptides and proteins are associated with porcine cryptorchidism, using MALDI-TOF MS, LC-MS/MS and western blotting. Peptidomics is useful for discovering novel peptide indicators. PMFs have been used as the signatures of pathological changes, and for diagnosis, drug treatment monitoring, etc., whereas PCA plots show the homogeneity of samples. Although the DT is in the scrotal sac at birth, with a similar environment to the NT, distinct clusters of NTs and DTs at 1–2 weeks of age indicated that both groups were not interchangeable. In our previous study, differential expression of androgen receptor was reported in DTs and NTs both at 1–2 weeks and at 12 weeks, supporting the notion that DT and NT conditions were not the same [6]. Peptidomics has been used to identify tumor-derived human leukocyte antigen-I (HLA-I) and HLA-II binding peptides from human tumors, which could be exploited further in precision cancer therapy [13]. It could also be used to show dairy product deterioration and to monitor the health status of cows [14, 15]. In pigs, peptidomes have been reported in brain tissue, gastropancreatic digestion of milk proteins, plasma after hepatectomy to search for peptide indicators of liver regeneration, and epididymal sperm maturation [16–19]. In our present study, we have discovered a number of novel peptides in NT and UDT (Fig. 1). These peptides should be studied further to improve our understanding of the pathogenesis of the disease and for molecular targeted therapy in the future.

In commercial pig farms, NTs can be collected only when the pigs are routinely castrated at the age of 1–2 weeks in farrowing pens and rechecked at the age of 12 weeks in nursery pens (after weaning). The protein data from NTs at the age of 1–2 weeks were used to study unique protein expression for UDTs and DTs at the same age. The data from NTs at the ages of 1–2 and 12 weeks were used to study unique protein expression for UDTs and DTs at the age of 6 weeks, as data from the age of 6 weeks were possibly covered by those from the ages of 1–2 and 12 weeks. TNFRSF18 expression was shown in UDTs at 15 weeks by LC-MS/MS and western blotting. In fact, TNFRSF18 has been reported to be either a pro- or anti-apoptotic inducer, depending on the tissue [20]. TNFRSF18 has been demonstrated to bind SIVA, a pro-apoptotic protein, and to induce apoptosis in the Cos cell line, but if TNFRSF18 bound TNF receptor-associated factor 2 (TRAF2), it would exhibit anti-apoptotic action [20, 21]. However, in our present study, we found a plausible relationship of TNFRSF18 with the apoptotic protein SIVA1 (Fig. 5a), and with caspases 3 and 8 via TRAF2 (Fig. 5b). The results of protein–protein interactions from STITCH corresponded with our previous study, which reported that high testicular apoptosis was shown in UDTs of cryptorchid pigs at 15–20 weeks of age [6]. In other studies, apoptosis has been found in UDTs more than in DTs of cryptorchid rats and mice [22, 23].

Cryptorchidism in pigs has been reported to be controlled by multigenes, and pig selection at the GGP and GP generations has been performed to eliminate sires with cryptorchidism or fathering cryptorchid piglets [24]. However, cryptorchid fattening pigs still appeared. Studying the potential signal pathway for TNFRSF18-associated apoptosis in UDTs would improve our understanding of the mechanism of the disease. For future work, the expression of other proteins, not found in NT groups, should be investigated to increase the precision of their detection (Supplementary Table S2). A limitation of the present study is that we did not have NT samples at 6, 15 and 20 weeks of age, as for UDT and DT.

## Conclusion

The present study revealed the PMF, PCA and a novel candidate associated with porcine cryptorchidism, TNFRSF18, at 15 weeks of age. The results of this study may improve our understanding of the pathogenesis of the disease.

## Materials and methods

### Animals

Twenty-four DTs (seven at 1–2 weeks, eight at 6 weeks, four at 15 weeks and five at 20 weeks of age) and 27 UDTs (ten at 1–2 weeks, eight at 6 weeks, four at 15 weeks and five at 20 weeks of age) were obtained from cryptorchid pigs on private farms in Thailand. Thirty-four NTs were collected from healthy pigs (fifteen at 1–2 weeks and nineteen at 12 weeks). Samples were collected when pigs were routinely castrated on the farms. After sample collection, pigs were clinically monitored until normal signs appeared, returned to the farmers and reared normally on the farms. The samples were obtained with the consent of owners, following the ethical guidelines required by the Chulalongkorn University Animal Care and Use Committee (CU-ACUC), Thailand (license number 1631016). Testicular tissues were kept in RNALater solution (Thermo Fisher Scientific, Waltham, MA, USA) at − 20 °C. Approximately 100 mg of tissue was cryogenically ground, followed by incubation in 0.5% SDS for 1 h at room temperature. Samples were centrifuged at 12000 rpm for 15 min. Supernatants were kept at − 20 °C until further analysis.

### Peptide analysis using MALDI-TOF MS

Samples were prepared as described previously [10] with some modifications. Briefly, a modified Lowry protein assay was used to measure total protein concentrations in the individual and pooled sample in each group [25]. Dried pooled samples were reconstituted in acetonitrile (ACN) with 5% (v/v) trifluoroacetic acid (TFA) before mixing with an equal volume of MALDI matrix (10 mg/mL α-cyano-4-hydroxycinnamic acid in 100% ACN containing 5% TFA). Sixteen replicates were spotted on to MALDI target plates (Bruker Daltonics, Billerica, MA, USA). Mass spectra were obtained using an Ultraflex III TOF/TOF instrument (Bruker Daltonics) in a linear positive mode within a mass range of 1000 to 20,000 Da. An external calibration was performed using a Proteo-Mass Peptide and Protein MALDI-MS Calibration Kit (Sigma Aldrich, St. Louis, MO, USA). A total of 500 laser shots at 50 Hz were used to generate each spectrum. Peptide mass spectra were processed using flexAnalysis v. 3.3 software, whereas the PMF and PCA of target mass spectra between 1000 and 20,000 Da were analyzed using ClinProTools v. 3.0 software [26–28]. The reliability and the accuracy of the candidate peak selection were evaluated by > 90% recognition capability and cross-validation values [28].

### GeLC-MS/MS-based protein identification

Samples were prepared as described previously [10]. Briefly, the concentrations of total proteins were estimated using a modified Lowry protein assay [25]. Protein samples in each group were pooled and 50 μg of pooled samples were separated by 12% SDS-PAGE (Atto, Tokyo, Japan). Gels were stained with Coomassie Brilliant Blue R-250 and then destained with 16.5% ethanol in 5% acetic acid before being scanned using a GS-710 scanner (Bio-Rad, Benicia, CA, USA). For in-gel tryptic digestion, 25 segments of proteins in each lane were digested in the gel with trypsin. Gel fragments were then cut into 1-mm^3^ pieces. Dehydrated samples were incubated with 10 mM dithiothreitol (DTT) in 10 mM NH_4_HCO_3_ to reduce disulfide bonds, and were alkylated in 100 mM iodoacetamide in 10 mM NH_4_HCO_3_ in the dark. After dehydration with 100% ACN, proteins were digested in-gel with 10 ng/μL sequencing grade modified trypsin (Promega, Madison, WI, USA) overnight at 37 °C. To extract peptides, gel plugs were incubated with 50% ACN in 0.1% formic acid (FA). Extracts were dried and stored at − 80 °C.

For LC-MS/MS analysis, peptide samples in 0.1% FA were applied to an Ultimate 3000 LC System (Thermo Scientific Dionex, Sunnyvale, CA, USA) using a PepSwift monolithic nanocolumn (100 μm internal diameter × 6.5 cm) at a flow rate 300 nL/min. A linear gradient of 10–90% ACN in 0.1% FA was prepared. The nanoLC system was coupled with an electrospray ionization (ESI)-ion trap MS (Bruker Daltonics). Raw data were converted into a software file format mzXML and quantified, using CompassXport software (Bruker Daltonics) and DeCyder MS differential analysis software (Amersham Biosciences, Little Chalfont, UK), respectively. For protein identification, the MS/MS data were searched against the NCBI mammal databases (downloaded June 2019), using MASCOT v. 2.2 software (Matrix Science, London, UK) as a database search engine. The following parameters were used for peptide assignation: trypsin as enzyme, one missed cleavage allowed, precursor charge of + 1, + 2 and + 3, mass tolerances of 1.2 Da for the precursor ion and 0.6 Da for the fragment ions, carbarmidomethylated cysteines as static modification, and methione oxidation as dynanic modification. Proteins were identified from one or more peptides with an individual MASCOT score corresponding to *P* < 0.05, and were classified according to their molecular function, biological process and cellular component using the PANTHER classification system, v. 8.1 [29]. Protein ID scores were obtained as the sum of highest ion scores for each unique peptide sequence. Relationships of protein sets among different sample groups were analyzed by a Venn diagram (http://bioinformatics.psb.ugent.be/webtools/Venn/) [30]. The association of selected proteins with apoptotic proteins, such as the apoptosis regulatory protein SIVA1, caspase 3 (CASP3) and CASP8, was analyzed using the STITCH database (http://stitch.embl.de) [31].

### Western blot analysis for validation of GeLC-MS/MS results

Ten micrograms of pooled protein samples were boiled at 95 °C for 10 min in loading buffer [0.5 M DTT, 10% (w/v) SDS, 0.4 M Tris-HCl pH 6.8 and 50% (v/v) glycerol]. Samples were separated using a pre-cast NuPAGE 4–12% (w/v) Bis-Tris gel (Thermo Fisher Scientific) and RunBlue MES Run Buffer (Expedeon, Heidelberg, Germany) at 100 V for 90 min. Gels were blotted on to Trans-Blot Turbo nitrocellulose membranes (Bio-Rad Laboratories, Hercules, CA, USA) at 25 V for 20 min in Trans-Blot Turbo 5× transfer buffer (Bio-Rad Laboratories). Total protein band intensities in each lane were detected by a Pierce Reversible Protein Stain Kit for Nitrocellulose Membranes (Thermo Fisher Scientific) according to the manufacturer’s instructions. After incubation with an Immobilon Block-CH chemiluminescent blocker (Merck, Darmstadt, Germany) at 4 °C overnight and washing with Tris-buffered saline containing 0.1% Tween 20 (TBST), primary antibody, 1:3000 rabbit polyclonal anti-human TNFRSF18 (ab10030, Abcam, Cambridge, UK), was applied at 4 °C overnight. After washing, membranes were incubated with 1:20000 horseradish peroxidase-conjugated goat anti-rabbit antibody (Abcam) at 25 °C for 1 h. The expressed proteins were visualized using ECL western blotting detection reagents (GE Healthcare, Chicago, IL, USA) according to the manufacturer’s instructions and were imaged with a ChemiDoc Touch Imaging System (Bio-Rad Laboratories). The immunoblot signals were analyzed by Image Lab 6.0.1 software (Bio-Rad Laboratories). Ratios of target protein band intensities to the total proteins in each lane were analyzed statistically as previously described [32, 33]. The western blotting was performed five times.

### LC-MS/MS for verification of expressed protein sequence

To verify TNFRSF18 protein identity, a membrane was incubated with Restore Plus Western Blot Stripping Buffer (Thermo Fisher Scientific) at room temperature for 15 min to remove antibodies. After washing with TBST, excised protein bands were incubated with 10 mM DTT in 10 mM ammonium bicarbonate overnight. Trypsin digestion was performed at 37 °C for 3 h, and protein samples were dried using a SpeedVac (Thermo Fisher Scientific). Proteins were reconstituted in 0.1% FA and applied to the LC-MS/MS as described above.

### Statistical analysis

To analyze intensity values of PMF spectra, three statistical tests [Anderson-Darling (AD), t-test/.

ANOVA (TTA), and Wilcoxon/Kruskal-Wallis (W/KW), incorporated into ClinProTools v. 3.0 software], were used. For the GeLC-MS/MS analysis, ANOVA incorporated into the DeCyder MS was used to identify significantly distinct peptide expression among sample groups. For western blotting, statistical analyses of protein expression data were conducted using GraphPad Prism v. 8.0.1 (GraphPad Software, La Jolla, CA). Statistically significant differential expression was analyzed by Kruskal-Wallis test with Dunn’s multiple comparisons test by comparing the mean ranks of each group with the mean rank of UDTs at 15 weeks of age. *P*-values less than 0.05 were considered to be statistically significant.

## Supplementary information

**Additional file 1: Supplementary Table S1.** Relative expression levels of divergent proteins in undescended testes in the abdominal cavity (UDT), descended testes in cryptorchid pigs (DT) and normal testes of healthy pigs (NT) at different ages, shown as log_2_ intensities

**Additional file 2: Supplementary Table S2.** Groups of proteins differentially expressed in undescended testes in the abdominal cavity (UDT), descended testes in cryptorchid pigs (DT) and normal testes of healthy pigs (NT) at different ages. Asterisks indicate groups, excluding the NT groups

**Additional file 3: Supplementary Fig. S1.** Representative total protein detection on nitrocellulose membrane. Lane 1, proteins from normal testes of healthy pigs (NT) at the ages of 1–2 weeks; lane 2, NT at the ages of 12 weeks; lane 3, undescended testes in the abdominal cavity (UDT) at the ages of 1–2 weeks; lane 4, descended testes in cryptorchid pigs (DT) at the ages of 1–2 weeks; lane 5, UDT at the ages of 6 weeks; lane 6, DT at the ages of 6 weeks; lane 7, UDT at the ages of 15 weeks; lane 8, DT at the ages of 15 weeks; lane 9, UDT at the ages of 20 weeks; lane 10, DT at the ages of 20 weeks.

## Data Availability

The datasets used and/or analyzed during the current study are available from the corresponding author on reasonable request.

## Abbreviations

3D-PCA: Three-dimensional principal component analysis
ACN: Acetonitrile
CASP: Caspase
DT: Descended testis
DTT: Dithiothreitol
GeLC-MS/MS: In-gel digestion coupled with mass spectrometry
HLA: Human leukocyte antigen
LC-MS/MS: Liquid chromatography–tandem mass spectrometry
MALDI-TOF MS: Matrix-assisted laser desorption/ionization time-of-flight mass spectrometry
NT: Normal testis
PMF: Peptide mass fingerprint
SDS-PAGE: Sodium dodecyl sulfate-polyacrylamide gel electrophoresis
TBST: Tris-buffered saline containing 0.1% Tween 20
TFA: Trifluoroacetic acid
TNFRSF18: Tumor necrosis factor receptor superfamily member 18
TRAF2: Tumor necrosis factor receptor-associated factor 2
UDT: Undescended testis
