# Supplementary figures and images for: Proteomic profiles of unilateral cryptorchidism in pigs at different ages using MALDI-TOF mass spectrometry and in-gel digestion coupled with mass spectrometry (GeLC-MS/MS) approaches
Source: BMC Vet Res. 2020 Oct 2;16:373. doi: 10.1186/s12917-020-02591-1 (PMC7532586; doi:10.1186/s12917-020-02591-1)

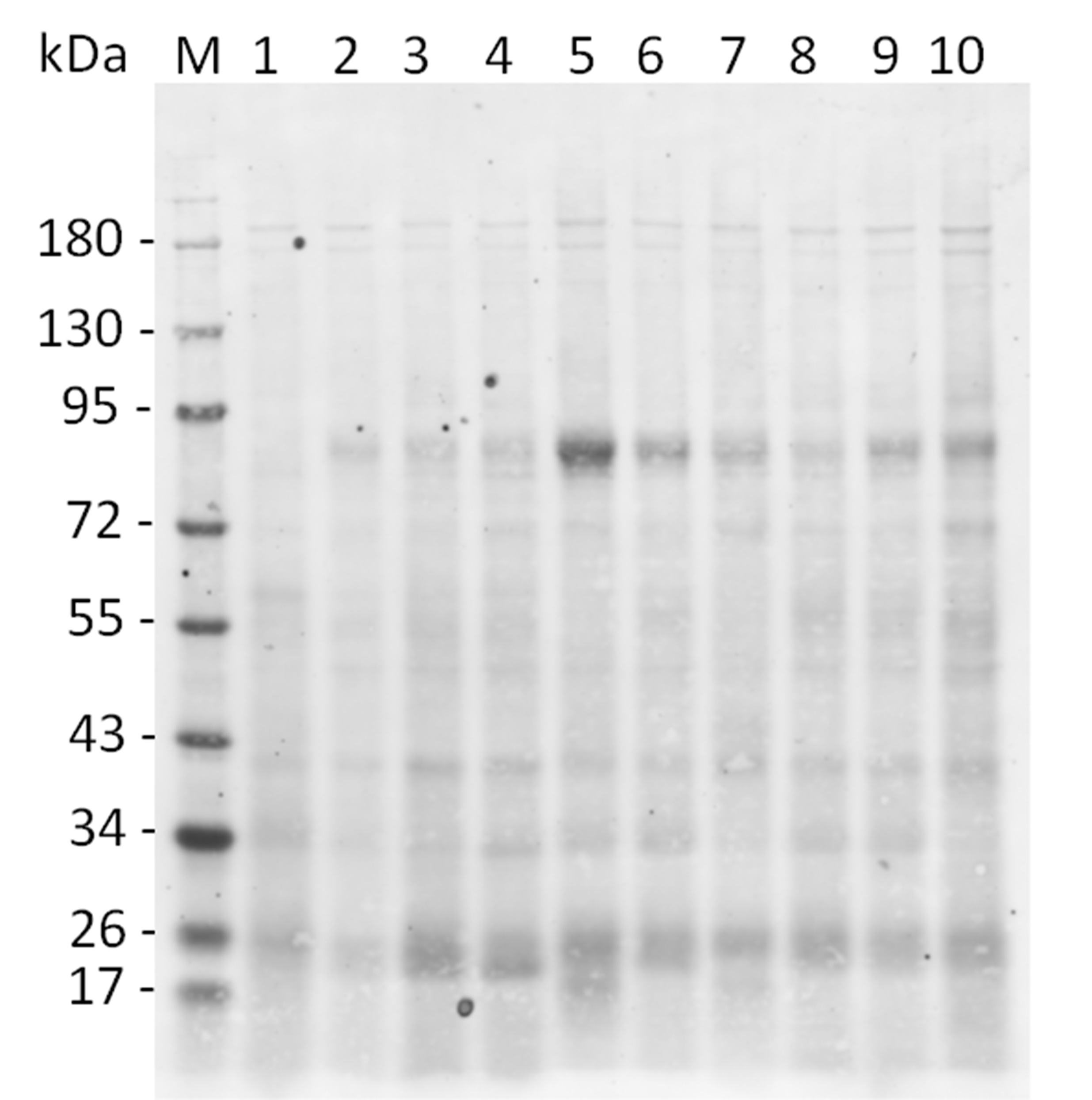

Supplement: Supplementary file 3 — Additional file 3: Supplementary Fig. S1. Representative total protein detection on nitrocellulose membrane. Lane 1, proteins from normal testes of healthy pigs (NT) at the ages of 1–2 weeks; lane 2, NT at the ages of 12 weeks; lane 3, undescended testes in the abdominal cavity (UDT) at the ages of 1–2 weeks; lane 4, descended testes in cryptorchid pigs (DT) at the ages of 1–2 weeks; lane 5, UDT at the ages of 6 weeks; lane 6, DT at the ages of 6 weeks; lane 7, UDT at the ages of 15 weeks; lane 8, DT at the ages of 15 weeks; lane 9, UDT at the ages of 20 weeks; lane 10, DT at the ages of 20 weeks. [file 12917_2020_2591_MOESM3_ESM.tif]
